# Quantifying viral load and characterizing virus diversity in wildlife samples with target enrichment sequencing

**DOI:** 10.1099/mgen.0.001513

**Published:** 2025-09-25

**Authors:** Laura Bergner, Stefano Catalano, Jenna Nichols, Ana Da Silva Felipe, Xiaofei Cao, Daniel Mair, Andrina Nankasi, Moses Arinaitwe, Alfred Mubangizi, Oliver G. Pybus, Claire Standley, Christina L. Faust, Jayna Raghwani

**Affiliations:** 1 School of Biodiversity, One Health and Veterinary Medicine, University of Glasgow, Glasgow, UK; 2 MRC–University of Glasgow Centre for Virus Research, Glasgow, UK; 3 Scotland Field Delivery, Animal and Plant Health Agency, Galashiels, UK; 4 Genetic Design and Engineering Center, Department of Bioengineering, Rice University, Houston, USA; 5 Vector Control Division, Ministry of Health, Kampala, Uganda; 6 Pathobiology and Population Sciences, Royal Veterinary College, Hatfield, UK; 7 Center for Global Health Science and Security, Department of Microbiology & Immunology, Georgetown University, Washington, DC, USA

**Keywords:** metagenomics, target-enrichment sequencing, virus transmission, virus load, virus diversity

## Abstract

Metagenomics is a powerful tool for characterizing viruses, with broad applications across diverse disciplines, from understanding the ecology and evolutionary history of viruses to identifying causative agents of emerging outbreaks with unknown aetiology. Additionally, metagenomic data contain valuable information about the amount of virus present within samples (i.e. viral load), which can provide insights into transmission potential, time since infection and, in turn, epidemic trajectories. However, before we can effectively use metagenomic data to inform transmission, we need to understand the general relationship between sequencing outputs and viral load. Here, using a commercially available probe panel targeting a wide diversity of viruses, we investigated the detection and recovery of virus genomes by spiking known concentrations of DNA and RNA viruses into wild rodent faecal samples. In total, 15 experimental replicates were sequenced with target enrichment sequencing and compared to shotgun sequencing of the same background samples. Target-enriched sequencing recovered all spike-in viruses at every concentration (10^2^, 10^3^ and 10^5^±1 log genome copies) and showed a log-linear relationship between spike-in concentration and mean read depth. Background viruses (including *Kobuvirus* and *Cardiovirus*) were recovered consistently across all biological and technical replicates and by shotgun sequencing, but genome coverage was variable between virus genera and likely reflected the composition of the target enrichment probe panel. Overall, our study highlights the strengths and weaknesses of using commercially available panels to quantify and characterize wildlife viromes and underscores the importance of probe panel design for accurately interpreting coverage and read depth. To advance the use of metagenomics for understanding virus transmission, further research will be needed to elucidate how sequencing strategy (e.g. library depth and pooling), virome composition and probe design influence viral read counts and genome coverage.

**Impact Statement**

There is an increased interest in characterizing and quantifying viruses in wildlife populations. We performed an experiment to determine if viral load could be quantified using target enrichment sequencing. We demonstrated that known quantities of viruses with diverse compositions and structures could be recovered and quantified with target enrichment sequencing. Additionally, we show that background viruses that are not directly in the probe panel can be recovered and quantified. This study provides validation for using target enrichment for not only describing virus species but also quantifying the viral load.

## Data Summary

The authors confirm that supporting data, code and protocols are publicly available or available within the article, supporting information and associated links. Code is available on GitHub https://github.com/cfaustus/te_ug_rodents. All raw sequencing data have been deposited in the Sequence Read Archive (SRR33126531-SRR33126549) under BioProject accession number PRJNA1250535.

## Introduction

Studying virus transmission in wild animal populations is inherently challenging [1]. In contrast to humans and livestock, where longitudinal passive surveillance is routinely used to monitor disease dynamics, similar approaches are often impractical in wildlife populations. In particular, virus detection in wild animals is likely to be hindered by the absence of overt or recognizable clinical symptoms in natural hosts, reduced mobility and shelter-seeking behaviours in sick animals and predation and scavenging of infected individuals [2].

Virus metagenomic (‘metaviromic’) surveys have greatly accelerated the discovery of new viruses in all kingdoms of life, significantly expanding our knowledge of global virus diversity [3,7]. However, despite containing quantitative information on virus abundance within samples, metaviromic data have yet to be fully exploited for investigating the ecological [8] and epidemiological dynamics of viruses. Viral load is an important metric for understanding onward transmission [9,10], identifying super-spreaders [11] and predicting clinical outcomes [12]. More recently, it has been shown that the distribution of virus quantitative PCR (qPCR) cycle threshold (Ct) values and viral reads within individuals can be used to understand population-level processes, such as epidemic growth rates and incidence [13,14]. Therefore, the ability to quantify abundance across a diversity of viruses would transform our ability to understand virus impacts on individuals and transmission within wildlife populations.

Traditionally, viral load is determined through antigen quantification, culture-based serial dilutions or qPCRs that use specific primers [15]. Characterizing viral loads across multiple viruses simultaneously has proved more difficult; optimizing multiplex PCR reactions requires time and resources and is not always possible for closely related viruses or under-characterized virus families [16]. Preliminary evidence suggests that metaviromics could help address this problem. For example, a comparison of qPCR Ct values with sequence read depth from individual-level host virome data from a mute swan population revealed a strong log-linear relationship, suggesting that read depth is a reliable proxy of viral load in wildlife [14]. Further support of the relationship between metrics of virus abundance in metaviromic data comes from controlled sequencing experiments, in which synthetic nucleotide sequences are added at known concentrations. These ‘spike-in’ experiments show a positive correlation between virus read abundance and depth and viral load [17,18].

One reason metaviromics remains underutilized for wildlife samples is that data generation is costly. Unbiased sequencing approaches often require high sequencing depth and can yield relatively small numbers of virus genomic data compared to background reads. One promising solution is target enrichment (also known as hybrid-capture) sequencing, which can simultaneously capture diverse virus genomes using custom-designed probe panels [19]. Target enrichment is well-suited for characterizing low-titre viruses and whole-genome sequences of multiple virus species within samples with high proportions of background reads (i.e. faecal samples and host tissues) [19,20]. Furthermore, this method tolerates significant sequence divergence from target probes (10–20 % [21]) and thus has fewer false negatives (robust to mutations at probe sites), is less likely to show uneven genome coverage and enables targeting of multiple sites compared to amplicon-based sequencing [22,23]. Custom probes have been used to characterize coronaviruses in bats [24,26], respiratory bacteria in chimpanzees [27] and influenza viruses from waterfowl habitats [28] and to undertake more general virus surveillance from environmental sources [29]. All the work to date has used custom-developed panels, increasing the time and expertise needed to execute these protocols. Increasingly, commercial probe panels are becoming available that can target a broad range of microbes and could be deployed in small batches. Commercial probe panels would allow rapid assessment of low-titre samples and require minimal troubleshooting, making them appealing for a diversity of research groups and sample types.

To understand the utility of target enrichment sequencing in quantifying and characterizing known and unknown viruses in wild animal populations, we set up a simple sequencing experiment where known concentrations of a mock viral community (comprising DNA and RNA viruses with diverse genome architectures) were spiked into a background matrix of RNA extracted from wild rodent samples. We also generated shotgun metagenomic data (i.e. without probe enrichment) from the same background samples. For spiked-in viruses (i.e. mock viral community), viral load was directly compared to read abundance and genome coverage. For viruses found in the background samples, the results were compared with data from shotgun sequencing and a custom qPCR. With this simple experimental design, we explored the benefits and challenges of using target enrichment sequencing to quantify and characterize viruses in wildlife populations.

## Methods

### Sample collection and RNA extraction

As a part of a wider field study, we captured small mammals with live humane traps in July and August 2022 in small-holder agricultural fields in eastern Uganda (Mayuge and Buvuma Districts) and humanely euthanized rodents and shrews via chloroform overdose (see ‘Ethics approval’). We collected faeces from the rectum during post-mortem examinations and stored samples immediately in DNA/RNA Shield (DRS) (Zymo Research Corp) at a 1:5 ratio by weight (e.g. 125 mg faeces in 500 µl DRS). Samples were stored initially at room temperature and then moved to −80 °C after a maximum of 38 days at ambient temperature. For this study, we used three pooled faecal samples (M1, M2 and M3); each containing four individuals of *Mastomys erythroleucus* and pooled in equal volumes (Table S1, available in the online Supplementary Material). We included a nuclease-free water control in RNA extractions, and the same negative control was processed for sequencing. We used the ZymoBIOMICS RNA Miniprep Kit (Zymo Research Corp) to extract RNA from 150 µl pooled faeces homogenate and eluted into 100 µl (more details in Supplementary Methods Section I). We measured RNA concentrations using the Qubit RNA HS Assay Kit on a Qubit 3 fluorometer (Thermo Fisher Scientific) and evaluated quality using an RNA 6000 Nano Kit on a 2100 Bioanalyzer (Agilent).

### Experimental design

**Fig. 1.**
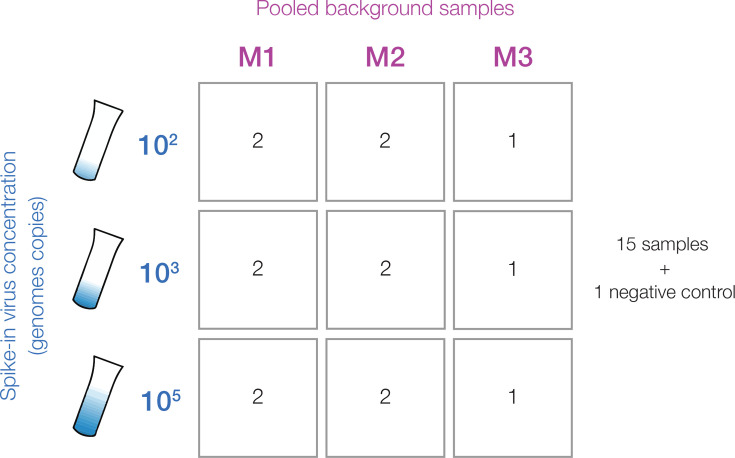
Schematic of the experimental design. Numbers inside each square represent technical replicates for each spike-in virus concentration (10^2^–10^5^ genome copies) and background sample (**M1–M3**).

We evaluated the performance of the Twist Comprehensive Viral Research Panel (CVRP) (Twist Bioscience) in detecting and quantifying spike-in (known) and background (unknown) viruses in pooled rodent faeces. The CVRP consists of 1,052,421 unique, 120 bp DNA probes that target 3,153 human and non-human associated viruses [30]. We selected a mock virome standard (Virome Nucleic Acid Mix; MSA-1008; American Type Culture Collection (ATCC)) that includes six viruses with diverse genome compositions and structures (human adenovirus 40, human betaherpesvirus 5, human respiratory syncytial virus, influenza B virus, mammalian orthoreovirus 3 and Zika virus; Table S2). To evaluate recovery of viruses present at different initial abundances, we prepared experimental samples composed of three different background sample pools (M1, M2 and M3) and three dilutions of mock virome (10^2^, 10^3^ and 10^5^±1 log genome copies) (Fig. 1). The spike-in concentrations chosen were based on a previous study evaluating sensitivity and specificity of the CVRP panel [17]. To evaluate protocol repeatability, we processed two technical replicates for each mock virome dilution in two background pools (M1 and M2), yielding a total of fifteen experimental samples (Table S3). Each background pool (M1-M3) was diluted to an initial concentration of 10 ng µl^−1^: 5 µl of this background was added to 5 µl relevant dilution of virome mix and 5 µl of nuclease-free water to achieve a concentration of 3.3 ng µl^−1^ background sample for protocols. To monitor contamination during library preparation, the nuclease-free water negative control from RNA extractions was processed alongside experimental samples.

### Library preparation for target enrichment sequencing

We prepared libraries using workflows for the Total Nucleic Acids Library Preparation EF v2.0 and Target Enrichment Standard Hybridization v1.0 Kits (Twist Bioscience). We followed all manufacturers’ instructions, except for modifications to first-strand cDNA synthesis for some samples (more details in Supplementary Methods Section II). We diluted experimental samples to a concentration of 3.3 ng µl^−1^ of background RNA and a volume of 15 µl. We performed first-strand cDNA synthesis using ProtoScript II (New England Biolabs) for samples with background M1 and M2, while SuperScript II (Thermo Fisher Scientific) was used for samples with background M3 and the negative control. We sequenced pools on an Illumina NextSeq 500 using a Mid Output 300-cycle cartridge, 2×150 bp with a target of 16M reads per sample at the MRC-University of Glasgow Centre for Virus Research.

### Library preparation for shotgun sequencing

We used Glasgow Polyomics’ sequencing service for shotgun sequencing. Samples were prepared using the QIAseq FastSelect Epidemiology Kits (QIAGEN) to remove rRNA and then the NEBNext® Ultra™ II Directional RNA Library Prep Kit for Illumina (New England Biolabs) to create libraries. They sequenced samples on an Illumina NextSeq 2000 using a P3 200-cycle cartridge, 2×100 bp with a target of 5M reads per sample.

### Quantification of background *Kobuvirus* using qPCR

To assess the capture efficiency of the CVRP for quantifying viruses present in the rodent sample background (i.e. viruses that were not spiked-in), we developed a qPCR assay for a rodent *Kobuvirus* detected in experimental samples (see ‘Results’). Primers and probes for the qPCR assay were designed for the polyprotein gene of the rodent *Kobuvirus* using the Integrated DNA Technologies (IDT) PrimerQuest tool and purchased from IDT (Table S4, Supplementary Methods Section III). We generated cDNA from 300 ng extracted RNA from M1 and M2 with the SuperScript III First-Strand Synthesis System (Invitrogen) and random hexamers, which was then stored at −20 °C.

We performed qPCR reactions on cDNA using IQ Supermix (BioRAD) and the newly designed primers/probe for the rodent *Kobuvirus* on an AriaMx Real-Time PCR Instrument (Agilent Technologies) with the following cycling conditions: 5 min at 95 °C, and 59 cycles of 5 s at 94 °C and 45 s at 60 °C. We used serial 10-fold dilutions of extracted plasmid DNA containing a positive control fragment to determine amplification efficiency (see Supplementary Methods Section III). We did not use template controls as negative controls, and we ran both experimental samples and positive controls in triplicate. We generated a standard curve with positive control dilutions from 10^−2^–10^−8^, which demonstrated that the qPCR had an acceptable *R*^2^ value of 0.997 and efficiency of 108%.

### Bioinformatics

Raw read data were processed using a modified metagenomic workflow [14,31], which included adapter removal, filtering reads with quality scores ≥30 and read length ≥45 bp using cutadapt 1.18 [32]. Cleaned paired-end reads were *de novo* assembled into contigs using megahit 1.2.8 [33]. Assembled contigs were classified taxonomically using diamond 0.9.22 [34] and the National Center for Biotechnology Information (NCBI) non-redundant database (downloaded December 2023). Viral contigs were identified based on *e*-value scores <10^−5^. To determine read depth and coverage of spike-in viruses, reads were mapped against reference genomes provided by ATCC using bowtie2 under the ‘very-sensitive’ mode [35]. Coverage and depth were calculated before and after removing PCR duplicate reads using the ‘depth’ function in samtools [36]. Read depth refers to the number of reads covering each site in the genome. Code is available on the public GitHub repository: https://github.com/cfaustus/te_ug_rodents,and raw read data from this study are available in the Sequence Read Archive under BioProject accession number PRJNA1250535.

For background viruses, assembled viral contigs from shotgun sequence data were used as reference genomes to obtain estimates of read depth and coverage for both the target enrichment and shotgun sequence datasets. The same bioinformatic pipeline was used to re-analyse published data from a study that spiked in the same mock virome into a synthetic human sample background and also used CVRP for target enrichment [18].

## Results

We examined the efficacy of a commercially available target enrichment probe panel in characterizing unknown viruses and in quantifying abundances of spike-in viruses at known concentrations in wild rodent faecal samples. On average, we obtained 10,667,076 (range 565,371–53,224,935) cleaned reads per sample, while the negative control contained 21,999 reads (none of which were viral contigs). Samples were processed in two hybridization pools. Pools were similar in sequencing depth [Pool(P)1 mean depth: 10,411,807 reads; P2 mean depth: 9,591,710 reads] (Fig. S1). Three experimental samples with the M3 sample background were excluded from further analyses, as these were processed differently during cDNA synthesis and lacked technical replicates (see ‘Methods’). Although not directly comparable due to the presence of spike-in viruses in target enrichment samples, as well as different library preparation and sequencing depth, shotgun sequencing of the two sample backgrounds yielded lower proportions of viral reads (M1: 1.2%; M2: 0.5%) compared to target-enriched samples with 10^2^ spike-in viruses (M1:2.7%; M2:5.6%) (Table S3).

### Target enrichment sequencing of spike-in viruses

**Fig. 2.**
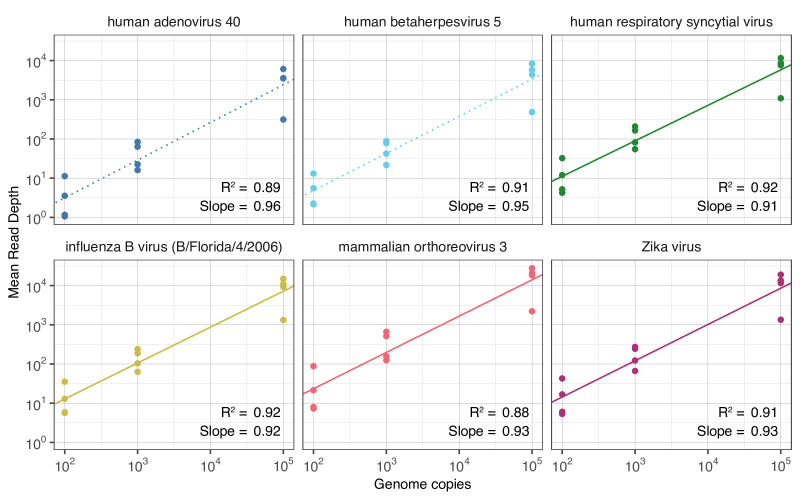
Relationship between spike-in viral load and mean read depth per virus. Deduplicated read depths (mean number of reads per site per genome) are shown for M1 and M2 backgrounds. Linear models are fit to virus species. Dotted lines are used for DNA viruses, and solid lines are used for RNA viruses.

We detected all six of the spike-in viruses in replicated sample backgrounds M1 and M2 and at each viral load (10^2^, 10^3^ and 10^5^) (Fig. 2). For each spike-in virus, we observed an increase in read count and read depth (number of reads per site) with increasing viral loads, which was consistent regardless of method used for normalization and whether reads were deduplicated or not (Fig. S2). Log-linear models showed a similar effect of viral load on mean read depth across viruses, with only minimal variation in the regression slope (range 0.91–0.95) and *R*^2^ (range 0.88–0.92) across viral taxa (Fig. 2). After normalizing for genome length, we observed relatively lower read depths for DNA viruses (*Adenovirus* and *Betaherpesvirus*) and similar read depths for RNA viruses, except mammalian orthoreovirus, which had higher read depth (Fig. S2). There was some evidence of variability between hybridization pools in terms of viral read count and mean read depth (Fig. S1). Trends in viral read count and the proportion of viral reads were relatively consistent between sample backgrounds (Fig. S3).

**Fig. 3.**
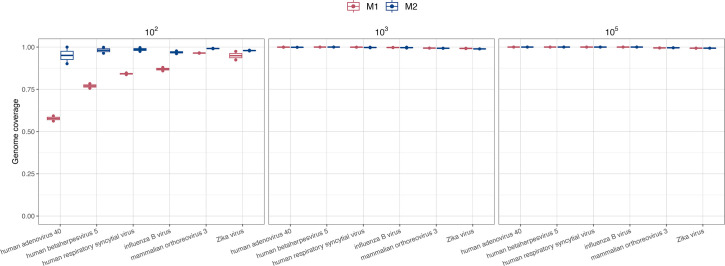
Genome coverage of spike-in viruses across spike-in viral loads. Boxplots are coloured by background sample (M1 or M2), and plots are arranged left to right with increasing spike-in loads.

Genome coverage increased with spike-in viral load (Fig. 3) . We recovered full genome coverage for all six viruses at higher viral loads (10^3^ and 10^5^) in all replicates. At lower concentrations, genome coverage was affected by virus species and sample background, with relatively lower coverage observed for DNA viruses and for the M1 sample background (Figs 3, S4–S9). Compared to the other spike-in viruses, human adenovirus exhibited lower read depth and more sparse genome coverage, particularly at lower viral loads (Figs 2, S2 and S4). To understand this observation, we re-analysed data from another study that also evaluated CVRP with the same mock virome standard [18], which similarly found lower depth and coverage for human adenovirus (Figs S10–S12).

### Target enrichment sequencing of background viruses

**Fig. 4.**
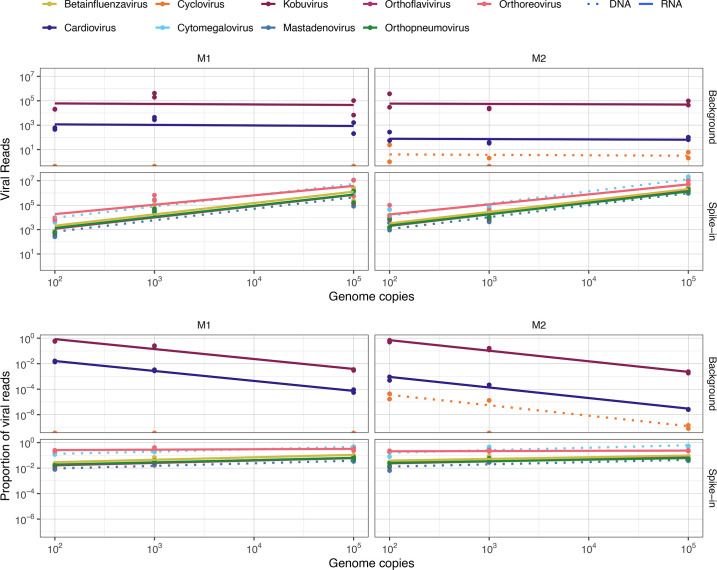
Recovery and read depth of spike-in and background viruses across technical and biological replicates. Lines are predictions from linear models fit to the genera. The linear fit to background viruses is poorer (i.e. more variation) compared to spike-in viruses for viral reads.

We detected several non-spike-in viruses in our samples, including two picornaviruses (genera *Cardiovirus* and *Kobuvirus*) and one circovirus (genus *Cyclovirus*). *Cardiovirus* and *Kobuvirus* were found in both sample backgrounds (Fig. 4) and in shotgun sequence data and are hereafter referred to as ‘background viruses’. *Cyclovirus* was detected in target enrichment and shotgun sequence data, although only short contigs were recovered (~400 bp). In target enrichment data, we observed lower read counts (Fig. 4), read depths (Fig. S13) and genome coverage for *Cardiovirus* than for *Kobuvirus* (Fig. S14). We found that reads were clustered in certain regions of the *Cardiovirus* genome for target enrichment sequencing, but not shotgun sequencing (Fig. S15).

The read count of background viruses did not notably vary with increasing spike-in viral loads (Fig. 4). However, the proportion of both total and viral reads, which include background viral reads, decreased as spike-in viral loads increased (Fig. 4). We further examined the impact of spike-in viral loads on background viruses by focusing on the rodent *Kobuvirus*, which was detected in all experimental samples (Fig. 4). Spike-in viral loads did not affect read depth and genome coverage, which were consistently high across all replicates (Figs S13 and S14). Instead, the sample background appeared to be more important in determining the overall genome coverage and coverage per site. Specifically, samples with M2 background consistently exhibited higher coverage for *Kobuvirus* in both target enrichment and shotgun sequence datasets (Figs S14 and S16). A similar but opposing pattern was observed for *Cardiovirus*, with coverage and depth generally higher in the M1 background (Figs S13-S15).

We used qPCR Ct values and read depth from shotgun metagenomic data to determine if *Kobuvirus* in target-enriched samples had comparable viral loads. Mean read depth was higher for M2 background than M1 in the shotgun metagenomic data (330.6 compared to 145 for deduplicated data), but there was no clear relationship between mean read depth and sample background in the target enrichment dataset (Figs 4 and S16). Compared to shotgun data, target enrichment samples had higher per-site coverage across samples in some genomic regions of the *Kobuvirus* (Fig. S16). qPCR data showed that the pools differed significantly in mean viral load (Welch t-test t=6.29; *P*=0.01), with lower Ct values indicating higher viral load for M2 (mean Ct 26.7±0.12) compared to M1 background (mean Ct 28.3±0.4).

## Discussion

Our study demonstrates that target enrichment sequencing can quantify and characterize spike-in and background viruses in wild rodent samples. Spike-in viruses were recovered at all tested concentrations, and we found a strong positive correlation between viral loads and mean read depth. Background rodent viruses were also enriched, but genome coverage was generally lower compared to spike-in viruses. Overall, our findings suggest that commercial target enrichment probe panels can be used to process wildlife samples, but custom-designed probes (i.e. probes designed to target specific virus species) are likely required for more precise quantification of viral load and higher genome coverage.

Although spike-in viruses were successfully recovered, mean read depth and genome coverage for certain viruses notably varied, particularly at lower viral loads. The variation in read depth between dsRNA, ssRNA and DNA viruses suggests that genome structure influences the efficacy of target enrichment, which has been previously observed by other studies [17,18]. This pattern could be related to cDNA synthesis and probes for both strands of dsRNA viruses. Differences in tiling coverage across viral genomes could also contribute to variation in capture efficiency between viral strains. The discernible lower depth and coverage of human adenovirus at low spike-in load is consistent with a previous study that evaluated Twist CVRP with ATCC virome mix [18], suggesting that this observation is unlikely to be caused by any laboratory or bioinformatic pipeline. Instead, it indicates some unknown interaction between the probe panel and spike-in adenovirus genome sequence. Altogether, our results highlight the importance of understanding the probe panel design, e.g. total number of probes, tiling density, relative concentration of probes across the virus genome, when evaluating viral loads with genomic data.

We recovered both DNA (*Cyclovirus*, ssDNA) and RNA (*Kobuvirus* and *Cardiovirus*, ssRNA) viruses from the background samples. Given that the CVRP primarily targets human viruses, the detection and coverage of background viruses will likely depend on the degree of sequence similarity to probes and how well the genetic diversity is represented by the probe panel. For example, while both *Kobuvirus* and *Cardiovirus* belong to the family *Picornaviridae*, differences in their detectability suggest that the genetic diversity of *Kobuvirus* is better accounted for by the probe panel compared to *Cardiovirus*. This is despite both genera having a similar number of virus representatives within the CVRP (*Kobuvirus*: *n*=31 compared to *Cardiovirus*: *n*=26). Additionally, the number of genomic sequences longer than 5,000 bp available in GenBank (accessed on 14 July 2025) from non-human hosts is substantially higher for *Kobuvirus* (*n*=340) than for *Cardiovirus* (*n*=80), indicating that *Kobuvirus* diversity is likely more comprehensively characterized. The higher genome coverage of *Cardiovirus* observed in the shotgun data further supports this idea and strongly suggests that the lower coverage of *Cardiovirus* in target enrichment data is likely due to probe mismatch. Both shotgun sequencing and qPCR demonstrated higher levels of *Kobuvirus* in M2 compared with M1, but similar mean read depths were observed among target-enriched sequencing replicates. This discrepancy may be due to the decision to equimolarize all background samples prior to target enrichment library preparation. Beyond the technical aspects of probe panel design, our findings underscore that we also need to carefully consider the workflow and composition of the probe panel when using this sequencing approach for virus discovery in wildlife samples.

Our study demonstrates that multiple viruses can be quantified simultaneously with target enrichment sequencing, which represents a huge potential in understanding wildlife virus communities. Virus genomes with matches in the probe panel (i.e. Influenza B) and divergent from the probe panel (i.e. *Kobuvirus*) were sequenced at read depths comparable to spike-in concentrations and evaluation of genome copy number through custom qPCR. In theory, at higher concentrations of viruses, there will be competition for probe hybridization and sequencing depth, which would complicate multi-species evaluation. However, we did not see any plateauing of mapped read depth at higher concentrations; this may be due to the limited serial dilutions (three) or that concentrations were not yet at saturation levels. The differences observed between DNA and RNA viruses suggest there may be predictable effects of genome structure on read recovery that could be taken into account.

While our study provides initial evidence that target enrichment can be used to quantify viral load in wildlife samples, additional experiments are needed to understand the limits of detection and appropriate workflows and to identify necessary controls to enable reliable interpretation across sequencing runs and diverse sample types. In the future, artificial spike-ins could be used to help normalize between individual samples and improve comparability of read depths. These spike-ins could be virus sequences from species unlikely to be found in the host, spiked in at known concentrations and representing a range of genome structures. Optimization of the composition and concentrations of spike-ins would require additional validation. Further experiments can also investigate the probe panel design, including how the composition and sequence identity of the custom probe panel affect observed sequencing data. These studies would help determine if interpreting viral reads should statistically account for observed variability in the future (e.g. number of probes per viral genome, genome composition and reads per pool). There is also scope for future work to evaluate how analytical choices, including methods for normalization, affect the outcomes and interpretability of these data.

## Conclusion

Our study further supports the use of viral reads from target enrichment sequencing to assess viral load. The ability to quantify viruses is essential for understanding processes that shape the temporal and spatial patterns of virus shedding in wildlife populations. These methods can be scaled to larger studies to map viral load across populations and ascertain when and where transmission risk is elevated. This approach is valuable for understanding virus circulation in natural populations and/or assessing zoonotic spillover risk.

## Supplementary material

10.1099/mgen.0.001513 Supplementary Material 1.

## Abbreviations

ATCC: American Type Culture Collection
Ct: cycle threshold
CVRP: Comprehensive Viral Research Panel
DRS: DNA/RNA Shield
NCBI: National Center for Biotechnology Information
qPCR: quantitative PCR
